# RNA-seq analysis for detecting quantitative trait-associated genes

**DOI:** 10.1038/srep24375

**Published:** 2016-04-13

**Authors:** Minseok Seo, Kwondo Kim, Joon Yoon, Jin Young Jeong, Hyun-Jeong Lee, Seoae Cho, Heebal Kim

**Affiliations:** 1 Interdisciplinary Program in Bioinformatics, Seoul National University, Kwan-ak St. 599, Kwan-ak Gu, Seoul, South Korea 151–741, Republic of Korea; 2 CHO&KIM genomics, Main Bldg. #514, SNU Research Park, Seoul National University Mt.4-2, NakSeoungDae, Gwanakgu, Seoul 151–919, Republic of Korea; 3 Division of Animal Products R&D, National Institute of Animal Science, #1500 Kongjwipatjwi-ro, Wansan-gu, Jeonju-si, Jeollabuk-do, 55365, Republic of Korea; 4 Animal Nutritional physiology Team, National Institute of Animal science, #1500 Kongjwipatjwi-ro, Wansan-gu, Jeonju-si, Jeollabuk-do, 55365, Republic of Korea; 5 Department of Agricultural Biotechnology, Animal Biotechnology Major, and Research Institute of Agriculture and Life Sciences, Seoul National University, Seoul 151–921, Republic of Korea

## Abstract

Many recent RNA-seq studies were focused mainly on detecting the differentially expressed genes (DEGs) between two or more conditions. In contrast, only a few attempts have been made to detect genes associated with quantitative traits, such as obesity index and milk yield, on RNA-seq experiment with large number of biological replicates. This study illustrates the linear model application on trait associated genes (TAGs) detection in two real RNA-seq datasets: 89 replicated human obesity related data and 21 replicated Holsteins’ milk production related RNA-seq data. Based on these two datasets, the performance between suggesting methods, such as ordinary regression and robust regression, and existing methods: *DESeq2* and *Voom*, were compared. The results indicate that suggesting methods have much lower false discoveries compared to the precedent two group comparisons based approaches in our simulation study and qRT-PCR experiment. In particular, the robust regression outperforms existing DEG finding method as well as ordinary regression in terms of precision. Given the current trend in RNA-seq pricing, we expect our methods to be successfully applied in various RNA-seq studies with numerous biological replicates that handle continuous response traits.

In the transcriptome analysis field, many analytical approaches were successful for identifying varying characteristics depending on the given conditions. Of these approaches, mRNA abundance is most widely utilized for detecting DEGs. While diverse mRNA measuring platforms such as serial analysis of gene expression (SAGE), microarray, and RNA-seq, have been employed by many, recent studies implied that RNA-seq is most suitable for profiling mRNA expression in terms of reliability^1^,^2^. Understandably, the progression of method development for RNA-seq analysis is similar to that of microarray analysis because the difference, between the two, solely depends on their measuring techniques. In other words, experimental designs are equivalent in both platforms. Accordingly, it is important to connect the developmental process of microarray analyses for developing RNA-seq method. In this light, the structure of RNA-seq experiments must be much more complex, as they consider more conditions and biological variations^3^. Unfortunately, only a small number of replicated samples are available in most RNA-seq experiments due to its high cost. Consequently, most of recent statistical methods and research designs focus on simplifying experimental design by minimizing number of replicates (or removing replicates altogether) and groups of interest. Although the necessity of technical replicates is said to be minor in RNA-seq^2^, biological replicates are imperative to consider the differences of each individual in certain biological phenomenon. Sooner or later, the number of replicates used will increase for more accurate estimation. In symbiotic fashion, more complex experimental designs with various factors can be utilized with the increase of replicates. Most notably, various quantitative traits, which can be applied to a statistical model (i.e. BMI or waist-hip ratio of Human) are frequently used for obesity related analyses, as representative measures, in several clinical studies on microarray^4^,^5^,^6^). Many studies have already performed quantitative trait based analysis such as genome-wide association (GWA) and expression quantitative trait loci (eQTL) analysis, to investigate causal relationships of variant-trait and variant-gene expression, respectively^7^,^8^,^9^. By these quantitative trait analyses, heritable gene expression changes can be detected but not non-heritable expression changes cannot be identified (i.e. gene expression change corresponding to the epigenetic and/or environmental effects^10^). For this reason, many RNA-seq studies still have focused on the relationship between gene expression and experimental conditions regardless of the genetic effects. Hence, the current trend in RNA-seq analysis is to convert quantitative traits as categorical variable based on specific cutoffs, which facilitates the analysis in some practical issues^11^,^12^,^13^. However, employing non-converted continuous variables is more advantageous than using converted variables; the greatest advantage is that no within-group phenotypic variation is lost. Despite this advantage, most studies are forced to convert the variables from continuous to categorical, using specific cut-off criteria, because of practical problems such as budget or absence of the golden standard method for quantitative traits on RNA-seq analysis. While many analytical methods such as *DESeq2*^14^, *limma voom*^15^, and *edgeR*^16^ compares gene expression between two or more conditions of RNA-seq data, trait associated test based approach is yet to be researched. Here, we define specific genes associated with a target related traits (quantitative type) as TAG. In general, the TAGs can be defined by the linear regression analysis, through an association test between continuous traits and mRNA expression.

By incorporating such traits, well-developed statistical methodologies for microarray data can be extended to RNA-seq analysis. Some minor adjustments, however, are required to consider RNA-seq specific distribution assumtions^17^. As a solution, statisticians showed that count-type distributions such as Poisson distribution or negative binomial distribution are viable for assumption of the gene expression in RNA-seq. Under these assumptions, Generalized Linear Model (GLM), with gene expression as response variable, is handled in *DESeq2* and *edgeR*. From this statistical model, the detected genes can be interpreted to have significant impact on gene expression between groups. However, in some cases, the interpretation becomes ambiguous when the explanatory variable could reasonably be considered as a response variable, such as BMI and milk yield; these traits are also an outcome of individual biological activity. In this light, it is reasonable to consider these traits as response variable on the model. A continuous trait would usually satisfy the normality assumption, when the number of biological replicates is large enough. Therefore, several well-developed statistical models, based on Gaussian distributed assumption, become applicable in RNA-seq.

In this study, we investigated the pros and cons of the association test based approach on the RNA-seq analysis. We used two types of real RNA-seq data: (1) Human obesity related dataset composed with 89 biological replicated samples, (2) Holstein’s RNA-seq data composed with 21 biological replications and their milk production related traits. Using these datasets, we illustrate that the linear models are applicable in RNA-seq data analysis for diverse experimental purposes as well as diverse species.

## Materials and Methods

### Descriptions for two employing RNA-seq data along with continuous traits and its group variable based on certain criteria

#### Human obesity related RNA-seq data

We used 89 RNA-seq samples (GSE50244) including their expression values and trait information: BMI, sex, and age. Based on the BMI, group variable was coded as over-weight (OW) (BMI ≥ 25) or normal (BMI < 25). Total 89 human pancreatic islet donors without Type 2 diabetes were sequenced by Illumina HiSeq 2000. Based on the hg19 reference genome, paired-end reads were mapped by TopHat v.2.0.2^18^. The mapped counts were measured by HTSeq^19^ based on the Refseq gene annotation and finally mapped 18,566 genes. Then, the raw counts were normalized as trimmed mean of M-values (TMM) normalization method through edgeR package implemented in R. In the previous studies, primary goal was to detect novel genes influencing glucose metabolism using this RNA-seq data. Two transcriptome analysis were successfully performed using this data^20^,^21^. Of these researches, one is related to identifying novel genetic variants influencing gene expression by quantitative trait loci (QTL). The other was to characterize correlated genes with HbA1c by Spearman’s correlation test. While some significantly correlated genes were detected in this study, an association test was not performed using this data. Although sex and age should be considered when detecting trait related genes, their effects were not considered as covariates on the model.

#### Holstein milk yield related RNA-seq data

In order to check applicability of association study in another species, we performed RNA-seq experiment using 21 Holstein samples. All animal procedures were approved (No. 2012-C-005) by the National Institute of Animal Science Institutional Animal Use and Care Committee (NIASIAUCC), Republic of Korea, and performed in accordance with the animal experimental guidelines provided by NIASIAUCC. We measured bovine trait information such as normalized milk yield derived from the Korea Type-Production Index (KTPI) at the National Institute of Animal Science (NIAS), parity, and lactation period in order to identify milk production related genes. In addition, milk yielding ability group was codded as high yielding group (HY) and low yielding group (LY) based on KTPI. More detailed sample descriptions can be found in previous research^22^. From the RNA-seq analysis, 13,570 bovine genes were detected for the 21 Holstein samples. We generated raw RNA-seq, processed count, and covariate data, which are available at GSE60575 in the GEO database. In addition, a more detailed experimental pipeline can be found in (Supplementary methods). The mapping rates and mapped counts were represented in Table S1. Concordant with the human dataset, the raw mapped counts were normalized by TMM normalization method.

### Statistical methods for analysing RNA-seq data

#### Identification of trait associated genes (TAGs) using ordinary linear regression

In general, the TAGs can be defined by the linear regression analysis, through an association test between continuous traits and mRNA expression. By incorporating such traits, well-developed statistical methodologies for microarray data analysis can be applied to RNA-seq analysis with minor adjustments. A main reason that statistical methods from microarray studies cannot be directly employed by RNA-seq studies, is that gene expression on RNA-seq do not follow the normal distribution. For example, the most popular parametric method, t-test, requires a normality assumption about the response variable. Over the years, researchers assumed count type distributions, such as RNA-seq expression, to follow Poisson distribution or negative binomial distribution. These assumptions can be used in Generalized Linear Model (GLM) with gene expression as response variable. Therefore, many studies focus on identifying the significant slope (difference of the mean considering standard error) among different groups using GLM models between two variables: gene expression and group. However, in some cases, the interpretation becomes ambiguous when the explanatory variable could reasonably be considered as a response variable, such as BMI and milk yield; because trait affected gene finding is the ultimate goal. Therefore, it is conceptually acceptable to consider quantitative traits as response and gene expression as explanatory variable. By switching places between trait and gene expression variable on the linear model, we can successfully utilize the ordinary linear model based methods that require the normal assumptions of response variable.


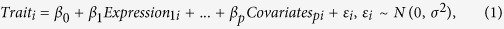


where 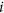 represents the individuals, *Expression*_*i*_ indicates the normalized gene expression value. *Trait*_*i*_ represents the each continuous trait such as BMI and milk yield. Covariate_*pi*_ represents covariate effects such as gender, age, and any considerable factors. From the model, the TAGs can be identified by t-statistic for each gene under the null hypothesis *H*_0_ : *β*_1_ = 0.

#### Robust methods for identifying TAGs using Huber’s M-estimator based robust regression

The ordinary least squared estimation (LSE) method is greatly affected by influential observations because it uses sum of squares. In general, probability of outliers in the biological data increases as the number of biological replications increases. Therefore, solving the outlier problem is unavoidable in the association based RNA-seq analysis. To build a robust estimator, Huber suggests robust regression^23^ using M-estimator. The M-estimator equation solves the following objective function:


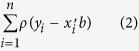



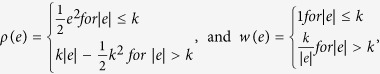


where *k* is called the tuning constant, 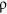 and *w*, are Huber’s objective function and weighted function, respectively. The main idea of robust regression method is to calculate weight of each point using iteratively reweighted least squares (IRWLS). Through a certain procedure, high-leverage observations have low weight and contribution to the fitted line. The weight depends on the k-value for the Huber method, usually *k* = 1.345σ is used. When tuning constant values are small, robustness increases for the outlier. However, non-influential points can be considered to be an outlier. In this study, we employed rlm package on R for calculating statistic for the fitted line and *f. robftest* function in the *sfsmisc* package for statistical test. The exact distribution and degree of freedom, of test statistics, in robust regression can be openly debated. In this study, to accomodate small sample size of RNA-seq data, wald-type inference for robust regression was employed^24^. In summary, three linear models such as ordinary, multiple, and robust regression were employed for detecting TAGs and they were compared by using GRACOMICS with their P-values^25^.

### Technical validation of significantly detected milk related TAGs using qRT-PCR

Real-time PCR was performed using QuantiTect SYBR Green RT-PCR Master Mix (Qiagen, Valencia, CA) and 7500 Fast Sequence Detection System (Applied Biosystems, Foster City, CA). Briefly, PCR was performed in a final reaction volume of 25 μL containing 200 ng cDNA, 12.5 μL SYBR Green RT-PCR Master Mix, and 1.25 μL of each of two primer solutions (10 μM). Parameters for thermal cycling were as follows: 50 °C for 2 m and 95 °C for 15 m followed by 40 cycles at 94 °C for 15 s, 60 °C for 30 s, and 72 °C for 30 s. ΔCt values were used to determine relative gene expression levels. All data was normalized with the housekeeping Ribosomal protein S9 (RPS9) gene.

### The simulation study using mock comparison to observe proportion of false discoveries in each approach

For investigation of false discoveries’ proportion, we followed the simulation setting of a recent study^26^. Four statistical approaches: *DESeq2*, *limma Voom*, ordinary regression, and robust regression, were compared with changing number of biological replication using mock comparison. In short, mock comparison is a method for estimating proportion of false discoveries. By permutating real group or trait, no significant result is expected in the mock comparison because test statistics are derived from the null distribution. The proportion of false discoveries were calculated in this fashion: Number of commonly identified genes in mock and real data divided by the Number of significant genes in real data. We repeated the test 1,000 times for each condition, for estimating standard errors of the false discoveries proportion. Through simulation, we calculated and compared the proportion of false discoveries of four methods: two suggesting approaches (Ordinary regression and robust regression) and two existing methods (*DEseq2* and *limma Voom*).

## Results

### Checking the validity of the normality assumption in the significant results from suggested association models

In order to show the suitability of ordinary linear model in RNA-seq, the required normality assumption of the residuals was first checked. The Shapiro-Wilk’s test^27^ was employed on the whole genes included in our two datasets. No significant genes were detected in both datasets under the significance level (FDR adjusted P-value < 0.05). Additionally, quantile-quantile plots (QQ-plot) of the 10 genes with lowest p-values (the worst cases) were drawn, as shown in (Figure S1) in order to visually check the normality. As a result, normality of the most genes is satisfied in both datasets.

### Detecting trait associated genes using ordinary linear regression

#### Human obesity related RNA-seq data

**Figure 1:**
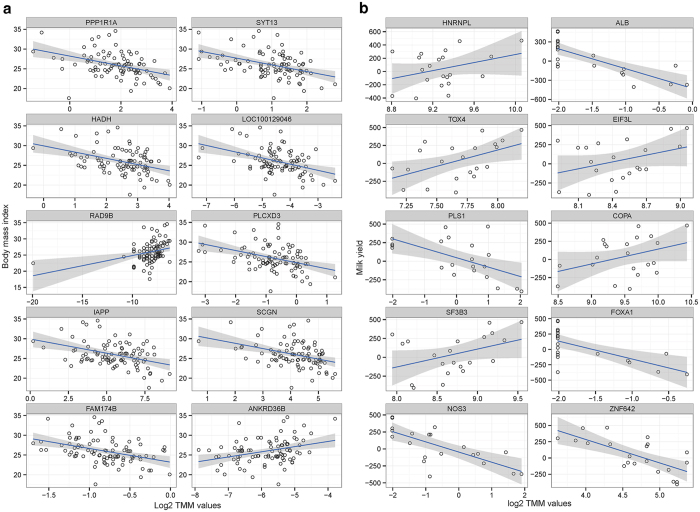
Ten of the most significantly associated genes from the simple linear regression with two types of RNA-seq data, respectively. The X-axis represents log2 TMM normalized gene expressions. Y-axis represents quantitative traits such as BMI and breeding value of the milk yield for human and bovine RNA-seq data, respectively. The blue-lines and grey-area represent estimated fit-line and standard errors estimated from the ordinary lease squared estimator, respectively. (**a**) Significantly detected BMI-associated genes in human RNA-seq data analysis (FDR adjusted P-value < 0.1). *RAD9B* gene shows that the result is strongly affected by outlier points. (**b**) Significantly detected milk yield-associated genes in bovine RNA-seq analysis. In the result of bovine analysis, relatively higher standard errors were estimated than human analysis, which may be due to the sample size difference.

The association tests were performed between BMI and gene expression using human RNA-seq. As a result, 30 genes were significantly detected in ordinary regression (FDR adjusted P-value < 0.1) (Table S2). The expression levels of those genes were highly associated with BMI as shown in Fig. 1a. As we employed simple linear regression, gene expression was only considered as explanatory variable on the model, and the results were analogous to the previous study^20^ (correlation test based approach). However, one of the advantages of employing regression model is that covariate adjustment is possible. In the obesity related studies, gender and age are highly influential factors, and they should be considered in the model (for these reasons, we performed multiple linear regression including gender and age terms on the model). As a result, 10 genes were significantly observed as BMI-associated genes when considering gender and age effects (FDR adjusted P-value < 0.1) (Table S3). In both models, *PPP1R1A* gene was found most significantly associated gene with BMI. In the Fig. 1a, negative association relationship was observed between *PPP1R1A* gene expression and BMI. While significantly detected genes showed that reasonable associated relationship with BMI, *RAD9B* was highly affected by outlier, which is limitation of the ordinary least squared method.

#### Holstein milk yield related RNA-seq data

For identifying milk yield-associated genes, regression analysis was applied into the 21 Holstein’s RNA-seq data. No significant genes were detected as milk yield associated gene (FDR adjusted P-value < 0.1). The main reason is that the sample size is not enough for detecting TAGs. We used a softer threshold, significance level as P-value < 0.05 without multiple testing adjustment, to determine milk yield related genes. We performed two linear models similar to that of human RNA-seq data above, simple and multiple linear regression. In the Holstein’s data, parity and lactation period should be considered as covariates because these factors are well-known variables that highly influence milk production^22^. The results show 338 and 289 TAGs were significantly detected in simple and multiple linear regression, respectively (Supplementary file 2). In Fig. 1b, 10 of the most significant genes were visualized as fitted-line plots. The *HNRNPL*, *TOX4*, *EIF3L*, *COPA*, and *SF3B3* illustrate positive association with milk yield. On the other hand, negative association was observed in the *ALB*, *PLS1*, *FOXA1*, *NOS3*, and *ZNF642* genes.

### Detecting trait associated genes using robust regression via Huber’s M-estimation

#### Human obesity related RNA-seq data

**Figure 2:**
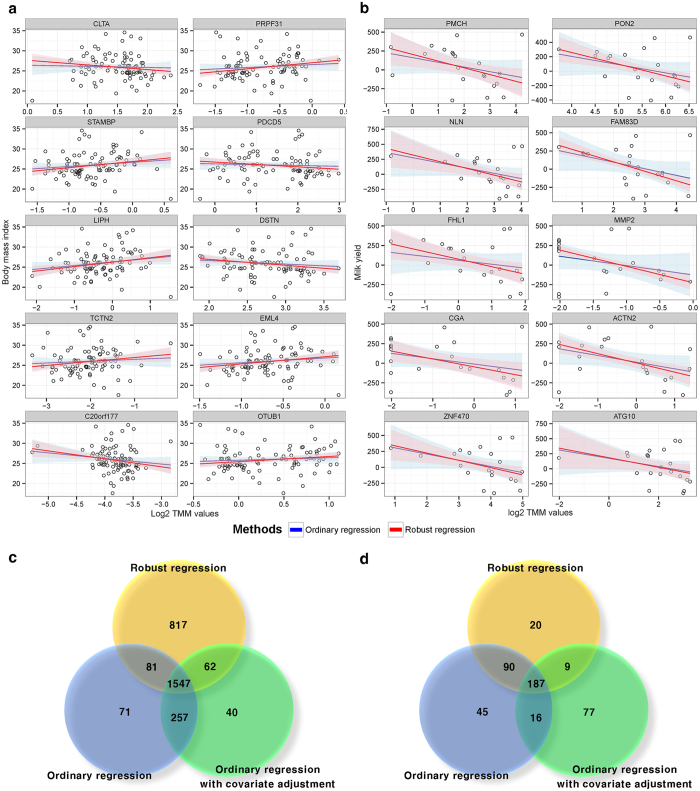
Identification of trait associated genes using robust regression adjusting for outlier effects. (**a**) The top 10 genes with a large difference between ordinary and robust regression, which were visualized as fitted plot from the analysis of human BMI related RNA-seq data. (**b**) Dramatically different top 10 genes were visualized as fitted plot in the bovine RNA-seq data. In the (**a,b**), blue and red lines represent fitted line in the ordinary and robust regression, respectively, and the standard errors represent same color with fitted line. (**c**) Venn-diagram for comparing TAG list in each model with human RNA-seq data. (**d**) Venn-diagram to compare significantly detected gene list in the bovine RNA-seq data analysis. In the (**c,d**), raw P-value < 0.05 is used as cutoff for comparison among different methods.

In the human RNA-seq data analysis, 932 genes were significantly detected as BMI associated gene (FDR adjusted P-value < 0.1) (Supplementary file 3). Compared to the results of the ordinary linear regression, relatively larger numbers of BMI associated genes were detected in the robust regression. In order to the visually check the differences between two methods, top 10 differentially expressed genes (based on the P-value difference) were visualized as fitted plots in Fig. 2a. A small discrepancy was observed between the two methods in terms of estimated slopes and standard errors. In Fig. 2c, numbers of significantly detected genes were compared among three statistical models based on their p-values (P-value < 0.05). As illustrated, a large number of detected genes were commonly identified among three methods. In addition, we observed that robust regression detects larger number of significant genes than ordinary regression by adjusting outlier effects.

#### Holstein milk yield related RNA-seq data

The robust regression was employed in bovine RNA-seq data for detecting milk-yield associated genes. As a result, 306 genes were significantly identified (P-value < 0.05). In Fig. 2b, unlike result of the human RNA-seq analysis, estimated fitted lines and standard errors were very different between two methods when the effects of outliers are adjusted. The *PMCH* gene is not detected significant in the ordinary regression (P-value: 0.429), but is detected highly significant in the robust regression (0.022). The rest of 9 genes also showed same patterns by reducing contribution of the extreme values for fitted-lines. In Fig. 2d, gene lists across the three linear models were compared to each other. Large number of commonly identified TAGs were observed between two regression methods, but robust regression makes less number of TAGs (306 genes) than ordinary regression (338 genes) in bovine RNA-seq analysis.

### Comparison between differentially expressed gene finding approach (using converted group variable using specific cut-off) and association test based approach

**Figure 3:**
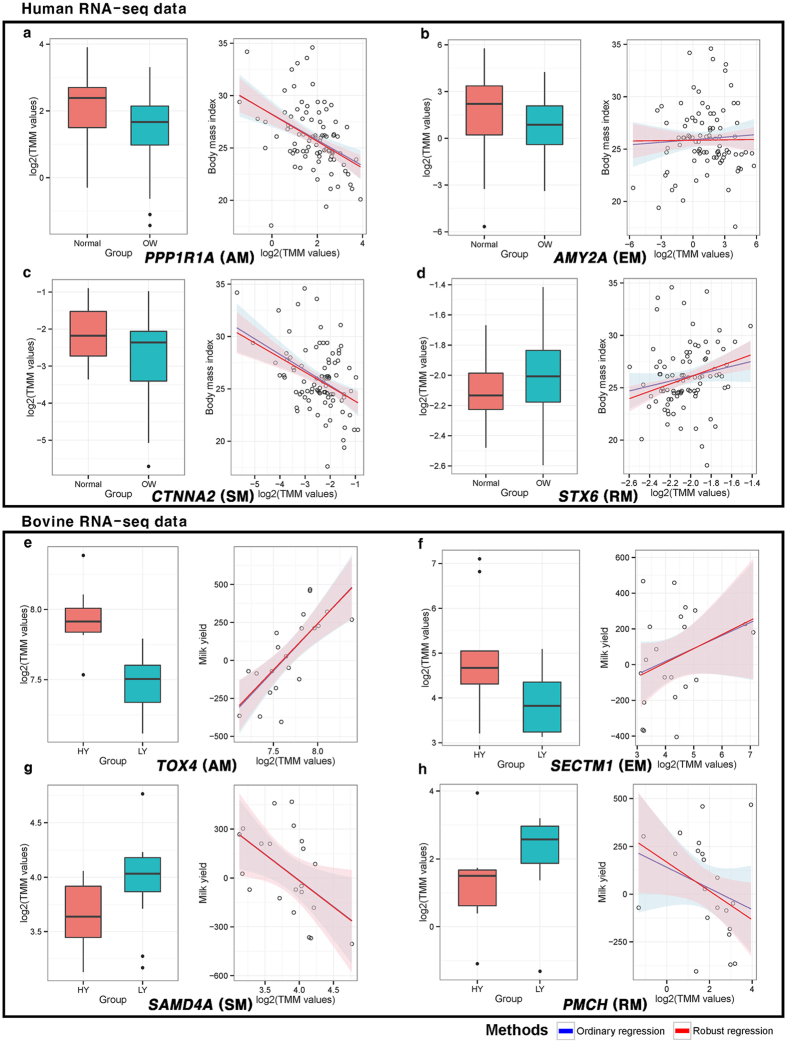
Representative patterns of gene expressions in four categories. Significantly detected genes were distinguished as four categories: ‘significant in all methods’ (AM), ‘only significant in existing methods’ (EM), ‘only significant in suggesting methods (SM), and ‘only significant in robust method’ (RM). The fitted line-plots are composed between TMM normalized values on x-axis and each trait on the y-axis. The blue and red lines represent fitted line of the ordinary and robust regression, respectively. The box-plots consist of forced conversion group such as normal vs. over weight (OW) for human data and high yield (HY) vs. low yield (LY) groups on x-axis and TMM normalized values on y-axis. In the result of human RNA-seq analysis, we selected four representative genes on their included category. (**a**) *PPP1R1A* gene that is significant from both linear based approaches and existing methods (DESeq2 based two group comparison). (**b**) *AMY2A* gene significant from two group comparison only. (**c**) *CTNNA2* gene from only the association test based approaches (**d**) *STX6* gene from robust regression method only. From the result of bovine RNA-seq analysis, four representative genes were selected such as (**e**) *TOX4* gene, significant in the all approaches. (**f**) *SECTM1*, only significant in the two group comparison. (**g**) *SAMD4A*, only significant in the association test based approaches. (**f**) *PMCH*, only significant in the robust regression.

One of the objectives in this study was to identify whether suggesting approaches are as suitable as existing methods (converted two group comparison based approach) for detecting candidate genes. In order to this, two approaches were compared; existing methods (*DESeq2* with group information) and suggesting methods (Ordinary regression and Robust regression with raw continuous variable). From these analyses, significantly identified genes were categorized as follows: ‘significant in all methods’ (AM), ‘only significant in existing methods’ (EM), ‘only significant in suggesting methods’ (SM), and ‘only significant in robust method’ (RM). Based on these categories, 4 significant genes were randomly selected for each of these categories in order to apprehend their characteristics. As Fig. 3 illustrates, distinct patterns are observed for each category. *PPP1R1A* and *TOX4* were significantly detected in the two group comparison based approach (P-values: 0.0001 and 2.82E-05) and association test based approach (1.27E-05 and 0.0013) as shown in Fig. 3a,e. These genes are not only differentially expressed among the two groups, but also strongly associated between gene expression and trait. *AMY2A* and *SECTM1* were detected significant from the approach of two group comparison only (0.001 and 0.0005 in the *DESeq2*) and (0.219 and 0.299 in ordinary regression). These genes show that although mean expression is different among two groups, no significant slope was observed as shown in Fig. 3b,f. Especially, we observed that existing method is very sensitive towards outlier values in the *SECTM1* gene. Unlike *AMY2A* and *SECTM1*, *CTNNA2* and *SAMD4A* was only detected significant in suggesting approaches (0.0002 and 0.0027) as shown in Fig. 3c,g. Finally, in Fig. 3d,h, the *STX6* and *PMCH* genes are only significant in the robust regression (0.002 and 0.0025). These P-values are summarized in Table S4, and the significant genes under 5% significance level are highlighted in red.

### The simulation result using mock comparison to observe proportion of false discoveries

**Figure 4:**
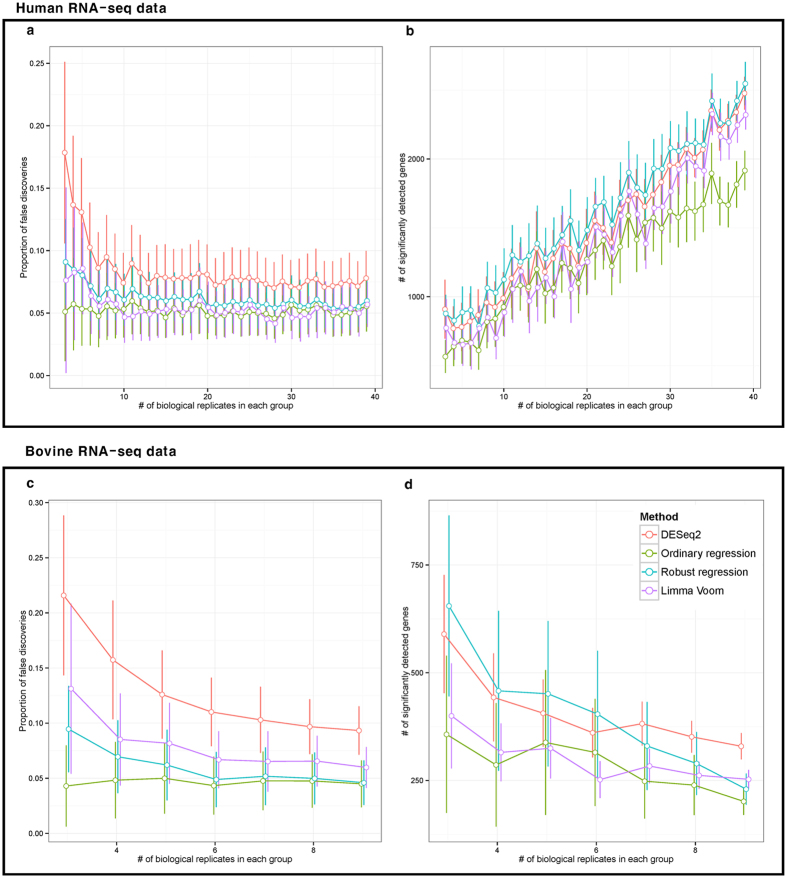
Calculation of the proportion of false discoveries using mock comparison. The result of proportion of false discoveries using mock comparison. As minimum number of replicates required for least square estimator (LSE) is 3 samples, 3 to 37 replicates in Human RNA-seq data and 3 to 9 replicates in bovine RNA-seq data in each group, were employed for mock comparison. The x-axis is number of biological replicate in each group and y-axis is proportion of false discoveries or number of significantly detected gene in each methods, (**a,c**) and (**b,d**), respectively, with 5% significance level. We observed suggesting approaches have smaller portion of false discoveries than existing methods especially in DESeq2.

In order to measure proportion of false discoveries in suggesting (Ordinary and robust regression) and existing methods (*DESeq2* and *limma voom*), mock comparison was performed. The outcome, of the simulation study using human data, suggests ordinary regression and *Voom* have smaller proportion of false discoveries compared to the robust regression and *DESeq2* as shown in Fig. 4a. In the figure, estimated proportion of false discoveries were similar in these three methods: robust regression, ordinary regression, and *Voom*, corresponding to the increase in number of biological replicates in each group. In Fig. 4b, in the three methods, we observed that number of significantly detected genes were increased as the biological replicates increased. Above all, *DESeq2* and robust regression detect a larger number of significant genes compared to the ordinary regression and *Voom*, in same conditions. From these results (Fig. 4a,b), although *DESeq2* can detect more significant genes than other methods, larger proportion of false discoveries was identified than the others. On the other hand, *Voom* and ordinary regression can detect comparatively smaller numbers of significant genes, but proportions of false discoveries are smaller than the others. Notably, robust regression show not only similar number of significantly detected genes as *DESeq2*, but also a lower proportion of false discoveries like the ordinary regression and *Voom*.

The mock comparison was conducted on the bovine RNA-seq data. Similar result patterns with that of human data were observed as shown in Fig. 4c,d. As a noteworthy difference, robust regression showed smaller proportion of false discoveries than *Voom* in Fig. 4c. In these figures, *DESeq2* and *Voom*, detect more genes than suggesting methods, at 5% significance level, regardless of the number of replicates, except in robust regression method. Considering the ratio between number of false discoveries and detected genes, robust regression has higher precision compared to other methods, and is much more practically applicable. In addition, ordinary regression has the lowest proportion of false discoveries but at the same time has the lowest number of detected genes; this method is most conservative in terms of precision. Since most RNA-seq experiments have small number of biological replicates, a robust regression is most applicable in terms of precision. As a result, linear and robust regression based approaches make satisfactory solution for identifying significant genes considering both the number of detected genes and proportion of false discoveries.

### Technical validation using qRT-PCR for verifying significance of the genes using bovine samples

From the results of the RNA-seq analysis and simulation, we observed that association test based approach is more reliable than two group comparison based approach that forces conversion of response to group variable, due to outlier handling and consideration of within-group biological variability. In order to revalidate this stability, a technical validation experiment was performed with randomly selected four genes, in each category, from the results of bovine RNA-seq analysis. Analysis results of these genes are summarized in Table S5. Using the selected genes, qRT-PCR experiment was performed with 23 biologically replicated samples. Table 1 shows the P-values of those genes resulting from a t-test between milk yielding ability groups, LY and HY, or linear regression with milk yield trait in the qRT-PCR experiment. The four genes belonging to the AM category are all significant in both types of statistical tests. In contrast, the genes included in EM category provide convincing evidence that they are false positives, because most of the test results suggest their insignificance. Compared to EM set, results in the SM and RM categories show smaller number of false positives, assuming the qRT-PCR results are valid. From these results, we propose that suggesting association test based approach can be found to be more reliable than two group comparison based approach in terms of false positives ratio.

### Biological interpretation for significantly detected TAGs

#### Human obesity related RNA-seq data

In human obesity related RNA-seq analysis, no significant genes were detected in the ordinary regression after multiple testing adjustment. On the other hand, 109 TAGs were significantly detected in robust regression (FDR adjusted P-value < 0.05). Of significantly detected genes, *PPP1R1A* (FDR adjusted P-value: 0.0228) is found most significant. In a previous study, *PPP1R1A* was also detected as a gene positively correlated with insulin secretion and negatively with HbA1c^28^. There was also a study which found 332 conserved pancreatic beta cell biomarker genes including *PPP1R1A* in Human and Rodent cells^29^. As a well-known fact, obesity and overweight are key risk factors for type 2 diabetes caused by pancreatic beta cells^30^,^31^. Another significantly detected TAG, *SCGN* (0.039) encoding Secretagogin, EF-hand calcium binding protein, has been reported as an islet-specific protein^32^. *ASB4* (0.039) encoding Ankyrin Repeat and SOCS Box Protein 4, was reported to be related to insulin secretion and action, energy metabolism, lipid biology, and/or adipogenesis^33^. Next, *DLL3* (Delta-like 3), notch ligands and member of notch signaling is detected significant (0.0396). Notch signaling as the central hub for glucose and lipid metabolism, was generally known to be involved in embryonic development, postnatal regeneration, and carcinogenesis of the liver^34^. And recently, there was a report that the inhibition of Notch signaling ameliorates obesity in mice^35^. Along with the above examples, there are many identified genes that were related to obesity. These literature reviews underpin that suggesting robust regression could be practically employed to detect obesity-related genes with BMI or other obesity related continuous traits.

#### Holstein milk yield related RNA-seq data

In the Holstein’s data, 16 technically validated genes were mainly focused. Of the validated genes, 10 genes were significantly associated with milk yield in Table 1. First, *NOS3* gene is already reported to be related with milk production, particularly in nipple erection in humans^36^. Because nipple erection is fundamental mechanism for milk production, *NOS3* gene would likely be a key marker for milk yield. Next, *HNRNPL* is significantly detected and validated, but, no evidence is reported to be related to milk production. This gene controls alternative splicing and mRNA transport from nucleus to cytoplasm^37^. In a previous study^38^, *HNRNPL* function is described as a specific activator of eNOS splicing, *NOS3* gene is identified in this research. Therefore, we suspect that *HNRNPL* to be also a strong candidate gene for controlling expression levels of milk production related genes. Next, we focus on TAGs only significantly identified in robust regression. As shown in Fig. 3h and Table 1, *PMCH* gene is only significant in robust regression. In mammals, *PMCH* encodes neuropeptides, melanin-concentrating hormone^39^; the hormone is linked to an increase in feed intake^40^,^41^. In addition, it has been reported that feed intake are related to milk yield^42^. From these reports, it can be suggested that *PMCH* is indirectly related to milk yield. In addition, deficit of milk production has been shown in the *KALRN* knock-out experiment^43^. This result is in keeping with our findings in profiling of cattle gene expression. The concordance with the previous literature reviews, about the detected and validated genes, indicate that suggesting association test based approach can be applied in bovine RNA-seq analysis as well as human RNA-seq analysis.

## Discussion

In this study, association test between continuous trait and gene expression was suggested for RNA-seq analysis. The proposed approaches were found more reliable than existing methods for the identification of trait related genes, as there is no loss of within-group variation information. In Figure S2, the reason behind small portion of false discoveries in the suggesting approach, is explained. This figure illustrates when forcing a conversion of continuous trait to group variable, consistent pattern information is easily lost and detected genes will have larger false discovery proportion. This phenomenon can be also observed in *CTNNA2* and *SAMD4A* genes, on Fig. 3c,g, which are only detected in suggesting approaches. When using two group information rather than continuous variable, variance of the individuals is neglected within a group. Its only focus is on the mean difference considering standard error; statistical investigation on linear tendency (constantly increasing or decreasing pattern) is neglected as shown in Figure S2. However, most researchers expect that significantly detected genes to linearly (or constantly) influence the quantitative traits. In addition, gene expression patterns of the *CTNNA2* and *SAMD4A* show insufficient mean difference among the forcibly converted groups, but these genes have steadily increasing gene expression patterns at raw quantitative trait state. Another advantage of suggesting approach can be found in *AMY2A* and *SECTM1* genes on the Fig. 3b,f, which ilustrated that two-group comparison approach is more sensitive to outliers’ effects. The simulation study (Fig. 4) supports these notions by showing that *DESeq2* have larger proportion of false discoveries than the suggesting approaches. Additionally, although qRT-PCR experiment was performed with small number of genes, the results underpin that suggesting approach are more reliable than existing methods. This number of genes (*n* = 4 in each category), in fact, are not enough for thorough calculation of false discoveries. Yet, qRT-PCR experiment is merely an additional evidence for simulation results. More accurate evidence about false discoveries is represented in simulation study using whole genes. However, all results provide supporting evidences that association test based approaches are more reliable in terms of false discovery proportion. Based on these evidences, we concluded association test can be practically considered and it can easily be added to the top choices for identifying TAGs on RNA-seq platform.

In this study, we considered several representative “control methods” to compare with the suggesting methods. In recent years, several methods have been introduced for detecting DEGs in RNA-seq such as Voom^15^, edgeR^16^, Cuffdiff2^44^, and SAMseq^45^. Specifically, some reported research showed that *Cuffdiff* results produce more false positives than *DEseq* and *edgeR*^46^. In case of *Voom*, a well-known tool for *limma* packages in microarray analysis, successfully released an extended RNA-seq version. *Voom* also uses a regression model, but it also only considers gene expression as response variable. Finally, *SAMseq* is a representative non-parametric method using permutation for detecting DEGs in RNA-seq. This method can apply detecting TAGs using quantitative traits in the linear model; however, with a satisfaction of appropriate assumptions, parametric methods are statistically more powerful. We have employed these popular tools, but observed more reasonably significant results with *DEseq2* when milk yielding group variable is analyzed.

We observed a gene which displays patterns of high difference between slopes of ordinary regression and of robust regression in Fig. 2 (See also Fig. 3d,h). For handling of outlier effects, we suggested robust regression; this method uses weight to reduce outlier points’ contribution to the fitted line, instead of discarding them upon statistical analysis. The outlier can be defined as two large groups; technical outlier and biological outlier. For the technical outliers (i.e. sequencing and/or align errors), analyzer entirely rely on technical replications. In RNA-seq, however, many studies are performed without technical replicates as measurements are much more precise in terms of measuring the transcriptome expression level compared to other platforms (i.e. microarray)^1^,^2^. A realistic solution, for situations where only biological replicates exist, is to detect the major pattern. Most biologists aim to detect significant TAGs, allowing some points to deviate from the major pattern because a large number of phenotypic traits are not Mendelian but are complex traits. In the complex traits such as obesity, milk-yield, and etc., single gene cannot completely explain phenotypic variation^47^,^48^. For this reason, generally, biological outliers exist in relationship between trait and gene expression. In this situation, association test is best choice for determining relationship between bio-marker and trait. However, ordinary regression is highly affected by outliers’ effects as seen in *SECTM1* gene (Fig. 3f), when calculating sum of squares for optimization of the fitted-line. Robust regression is suitable in this case, which is confirmed by our experiments. According to Fig. 3h, *PMCH* gene is only significant in robust regression, and this gene is successfully validated by qRT-PCR as shown in Table 1. Furthermore, *NLN* and *RECQL* genes included in the RM category, are also validated in qRT-PCR experiments. The simulation study reveals robust regression not only detects large number of significant genes, similar to two group comparison based approach, but also makes small proportion of false discoveries, as ordinary regression does. Therefore, results of our experiment reveals that robust regression is most suitable method for detecting TAGs in terms of precision.

Up to this date, regression based bio-marker detection was widely researched, particularly in genome study. With DNA markers, such as single nucleotide polymorphism (SNP), association study was already widely used (i.e. genome-wide association study (GWAS)). Based on our results, we expect that association study will be widely utilized in RNA-seq with diverse quantitative traits and numerous biological replications for detecting TAGs. Although association test is a strong tool for detecting TAGs, there are some limitations. First, in order to perform model-based approaches, numerous biological replicate samples are required because of statistical assumptions, especially in ordinary regression. Statistically, if number of samples goes over a certain threshold, sample arithmetic means follow normal distribution by central limit theorem (CLT). RNA-seq experiments are gradually becoming more affordable, which will lead to a higher number of biological replicates and allow more diverse conditions into consideration, and eventually satisfy requirements for minimum number of biological replicates. In our simulation study with 89 biologically replicated human samples, ordinary and robust regression showed that proportion of false discoveries is converged to 0.05 in Fig. 4. In this simulation, we assumed that the whole sample represent the population, which means that proportion of false discoveries is false positives based on such assumption. In this perspective, approximately 20 samples (10 biological replications in each binary group) were required for 5% significance level in terms of type-I error control in Fig. 4a. As for the other limitation, suggesting approaches need quantitative traits. Many RNA-seq based DEG analyses have restrictively compared two or more groups; only a few applications for observing significant genes using quantitative trait is proposed in RNA-seq study. For example, body size can be defined by height and waist circumference instead of ‘small’ and ‘big’ group variables^11^,^12^. Also, in the research of finding significantly related genes for drug dosage, degree of obesity hormone density, etc., raw quantitative traits can be utilized instead of converted group variables. Two group comparison based approach (i.e. equal variance t-test and *DEseq2*) are also one of the association test considering group variable as explanatory variable. However, in some cases, group variable should be considered in response variable similar to our employed dataset (degree of obesity and milk production). These variables could be considered as response variables on a statistical model, which is theoretically suitable because the milk production and degree of obesity are an outcome of gene activity.

In summary, we successfully demonstrated the application of the association test based RNA-seq analysis in order to identify trait associated genes (TAGs) by using human and bovine data. These results reveal that suggesting approaches outperformed that of *DESeq2*, in terms of proportional false discoveries. Moreover, robust regression is superior in terms of precision over *DESeq2* as well as ordinary regression. Given our study results, we anticipate other researchers conducting RNA-seq studies in the future to employ quantitative trait with sufficient number of biological replicates when exploring the TAGs.

## Additional Information

**Accession codes:** Human and bovine RNA-seq data are available at GSE50244 and GSE60575 in the GEO database, respectively.

**How to cite this article**: Seo, M. *et al.* RNA-seq analysis for detecting quantitative trait-associated genes. *Sci. Rep.* **6**, 24375; doi: 10.1038/srep24375 (2016).

## Supplementary Material

Supplementary File

Supplementary Data

## Figures and Tables

**Table 1:** The significant genes from the four categories and their P-value from qRT-PCR experiment.

Gene symbol	T-test	Linear regression	Category
HNRNPL	1.53E-02*	1.24E-02*	Significant results from all methods (AM)
NOS3	1.33E-02*	1.40E-02*	
SPTSSB	3.22E-02*	4.39E-03*	
TOX4	3.66E-03*	1.24E-02*	
FAM166B	5.01E-01	9.43E-01	Only significant results from existing methods (EM)
RNPC3	5.06E-02	1.00E-01	
SECTM1	3.01E-03*	5.03E-02	
SPESP1	2.43E-01	1.02E-01	
C25H16orf88	1.35E-02*	4.27E-02*	Only significant results from suggesting methods (SM)
KALRN	2.98E-02*	1.15E-02*	
PRIM2	4.27E-02*	9.67E-02	
SLC4A1	1.34E-02*	3.19E-03*	
NLN	1.71E-02*	1.85E-02*	Only significant results from robust method (RM)
PBX4	1.20E-01	3.68E-01	
PMCH	2.44E-02*	5.83E-03*	
RECQL5	8.72E-02	6.50E-03*	

*Significant result under the 5% significance level.
