# Supplementary material for: RNA-seq analysis for detecting quantitative trait-associated genes
Source: Sci Rep. 2016 Apr 13;6:24375. doi: 10.1038/srep24375 (PMC4829873; doi:10.1038/srep24375)
Supplement: Supplementary File [file srep24375-s1.doc]

# RNA-seq analysis for detecting quantitative trait-associated genes

Minseok Seo**^1,2^**, Kwondo Kim**^1,2^**, Joon Yoon**^1^**, Jin Young Jeong**^4^**, Hyun-Jeong Lee**^1,4^**, Seoae Cho**^2^**, and Heebal Kim**^1,2,3*^**

^1^Interdisciplinary Program in Bioinformatics, Seoul National University, Kwan-ak St. 599, Kwan-ak Gu, Seoul, South Korea 151-741,Republic of Korea.

^2^CHO&KIM genomics, Main Bldg. #514, SNU Research Park, Seoul National University Mt.4-2, NakSeoungDae, Gwanakgu, Seoul 151-919, Republic of Korea.

^3^Department of Agricultural Biotechnology, Animal Biotechnology Major, and Research Institute for Agriculture and Life Sciences, Seoul National University, Seoul 151-921, Republic of Korea.

4 Animal Nutritional physiology Team, National Institute of Animal science, #1500 Kongjwipatjwi-ro, Wansan-gu, Jeonju-si, Jeollabuk-do, 55365, Republic of Korea.

*[corresponding autho](mailto:corresponding author@email.example)r

Heebal Kim : Tel: +82-2-880-4803, Fax: +82-2-883-8812 ; E-mail : [heebal@snu.ac.kr](mailto:heebal@snu.ac.kr)

# Figure S1. Normality checking for investigating a model adequacy using quantile-quantile plot.


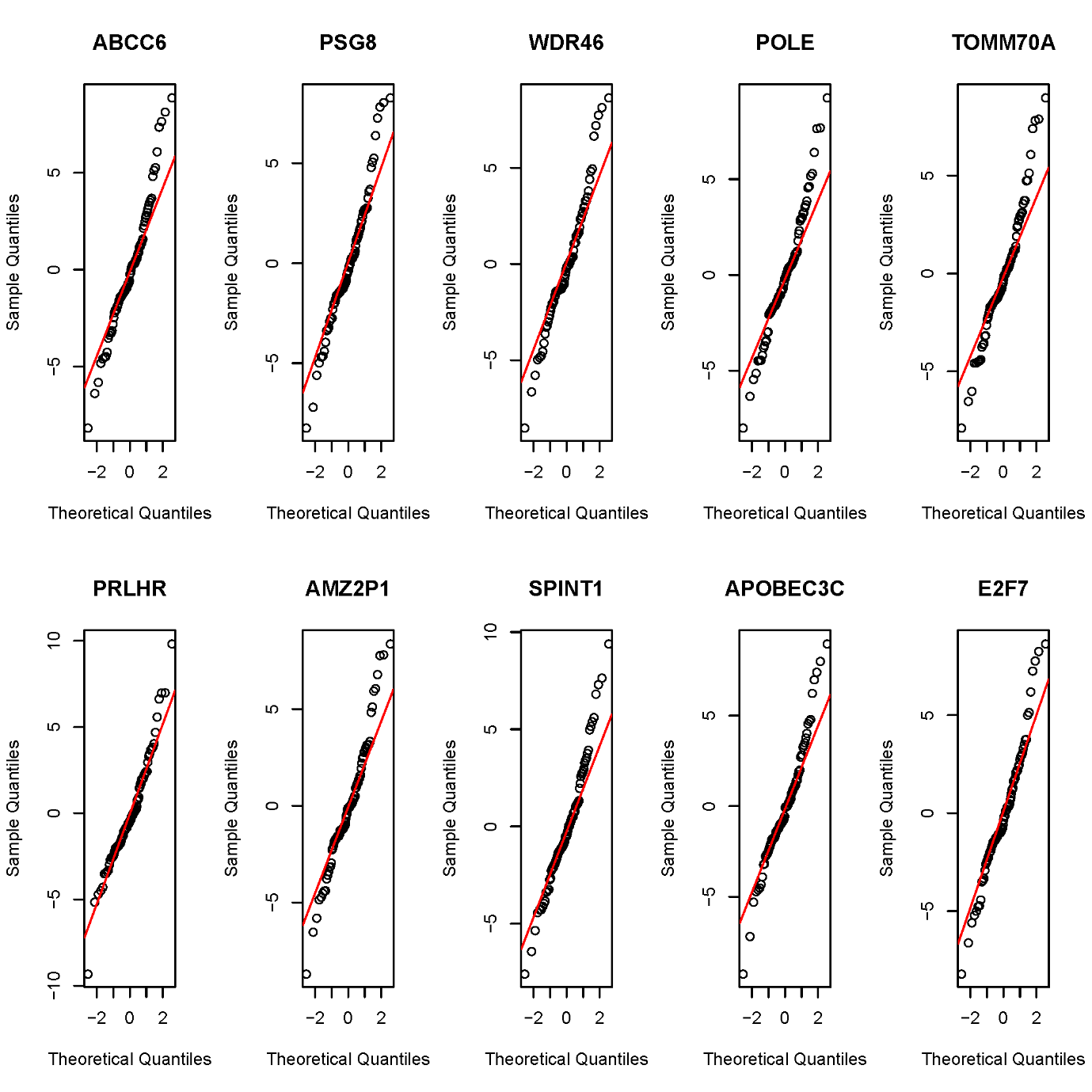


[Response variable: BMI]


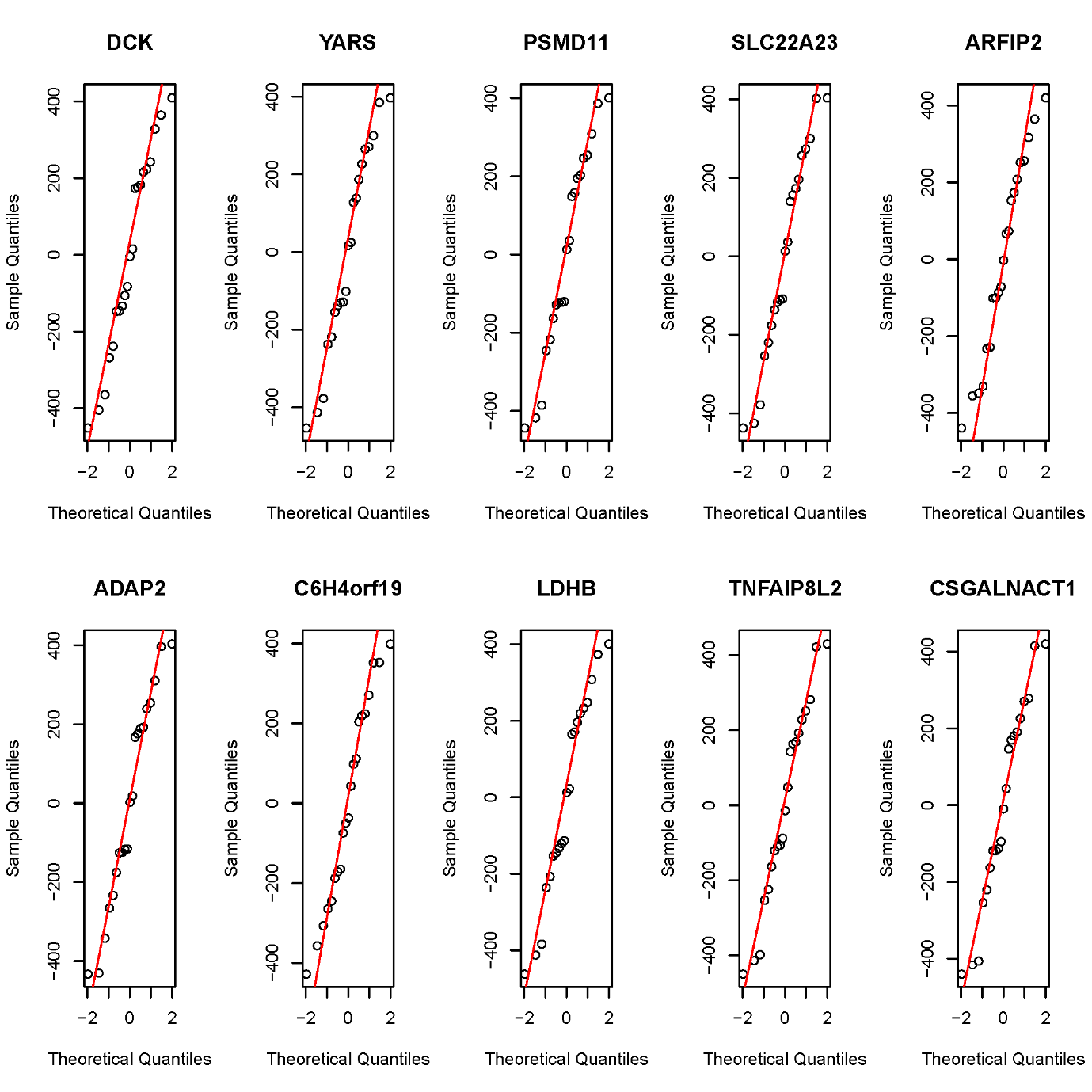


[Response variable: Milk yield]

# Figure S2. Reason behind smaller portion of false discoveries in suggested approached compared with two-group based approach.


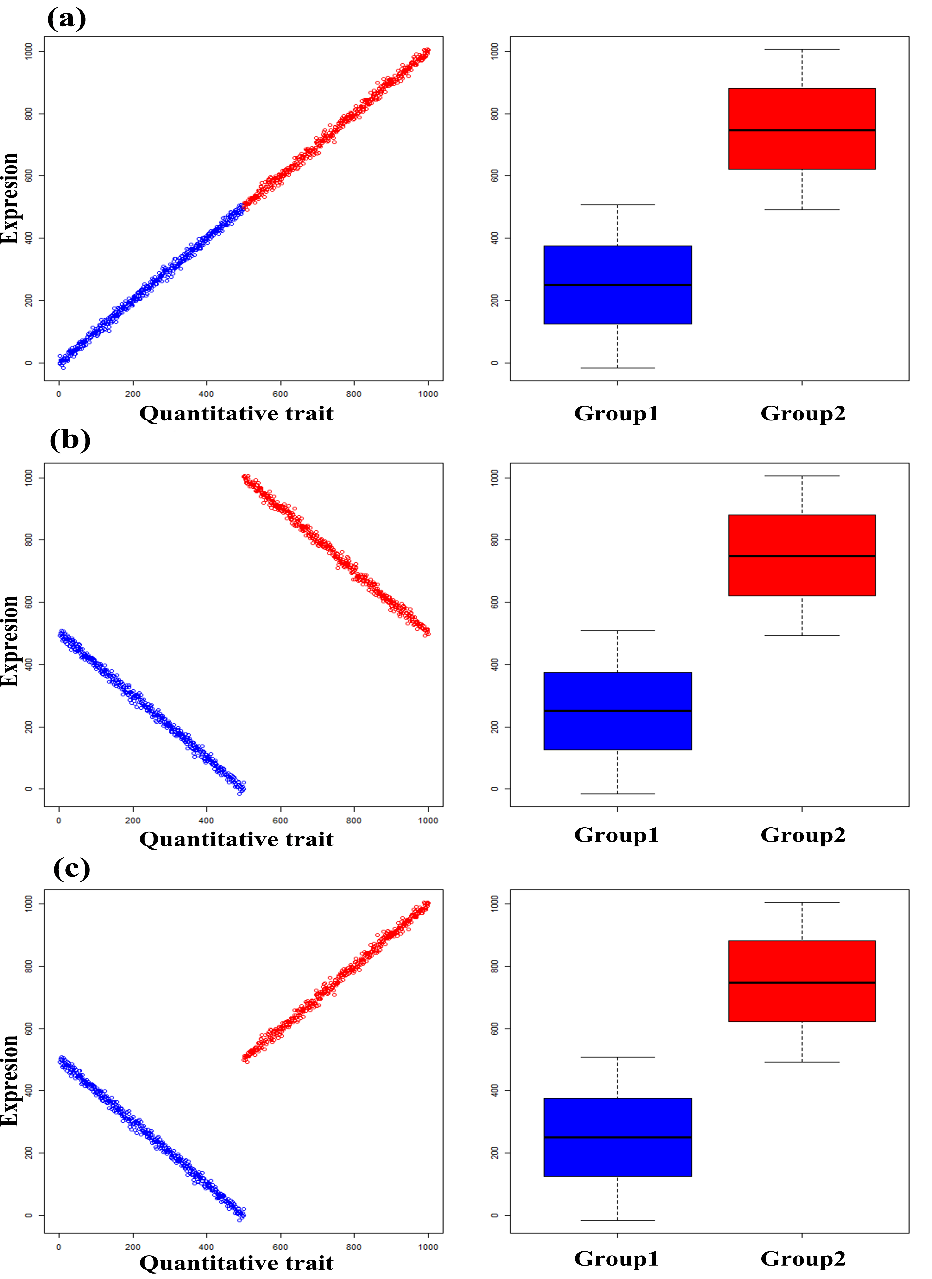


The example figure show why employing continuous type variable is more advantageous than using two group information. When using two group information rather than continuous type variable, within group variance is neglected. However, by using quantitative trait, we can achieve additional information such as linear tendency, constantly increasing or decreasing pattern.

Table S1. Mapping rates and number of mapped reads for Holstein RNA-seq experiment.

| **ID** | **Mapping_rate** | **# of Mapped Reads** |
| --- | --- | --- |
| **2814** | 77.40% | 43,980,082 |
| **2864** | 77.40% | 31,647,809 |
| **2893** | 75.50% | 32,808,428 |
| **2920** | 89.90% | 63,939,037 |
| **3014** | 79.60% | 29,940,147 |
| **3057** | 75.50% | 31,788,518 |
| **3064** | 79.40% | 48,398,676 |
| **3075** | 78.10% | 23,778,827 |
| **3135** | 79.80% | 29,346,535 |
| **3161** | 77.30% | 36,121,527 |
| **3198** | 77.80% | 22,507,466 |
| **3235** | 78.40% | 22,981,363 |
| **3262** | 76.20% | 55,247,146 |
| **3264** | 79.00% | 56,960,979 |
| **3272** | 89.00% | 71,257,880 |
| **3280** | 76.20% | 24,330,245 |
| **3369** | 78.80% | 24,840,297 |
| **3407** | 89.50% | 96,633,987 |
| **7598** | 76.50% | 31,647,259 |
| **7628** | 88.30% | 72,382,289 |
| **7643** | 86.10% | 67,969,067 |

Table S2. Significantly detected 30 obesity related TAGs in ordinary regression (FDR adjusted P-value < 0.01)

| **Gene_symbol** | **ID** | **Chromosome** | **FDR_M1** |
| --- | --- | --- | --- |
| **PPP1R1A** | P8750 | chr12 | 0.061471 |
| **SYT13** | P18875 | chr11 | 0.061471 |
| **HADH** | P8210 | chr4 | 0.075093 |
| **IAPP** | P6894 | chr12 | 0.075093 |
| **LOC100129046** | NR_034091 | chr1 | 0.075093 |
| **PLCXD3** | P5584 | chr5 | 0.075093 |
| **RAD9B** | P6433 | chr12 | 0.075093 |
| **ANKRD36B** | P24185 | chr2 | 0.084128 |
| **FAM174B** | P16809 | chr15 | 0.084128 |
| **PPM1E** | P5304 | chr17 | 0.084128 |
| **SCGN** | P27914 | chr6 | 0.084128 |
| **CTNNA2** | P13175 | chr2 | 0.086277 |
| **DNMT3B** | P8867 | chr20 | 0.086277 |
| **FAM105A** | P18727 | chr5 | 0.086277 |
| **KBTBD6** | P13069 | chr13 | 0.086277 |
| **LIPN** | P424 | chr10 | 0.086277 |
| **LYSMD2** | P15155 | chr15 | 0.086277 |
| **PCSK1** | P10665 | chr5 | 0.086277 |
| **PDX1** | P24068 | chr13 | 0.086277 |
| **PGM2L1** | P4721 | chr11 | 0.086277 |
| **TMCC3** | P18825 | chr12 | 0.086277 |
| **LOC255167** | NR_024423 | chr5 | 0.086408 |
| **KRT75** | P5426 | chr12 | 0.090524 |
| **SLCO1A2** | P27099 | chr12 | 0.090524 |
| **SLC2A2** | P16257 | chr3 | 0.093402 |
| **B3GALT2** | P13907 | chr1 | 0.094385 |
| **OXGR1** | P13684 | chr13 | 0.094385 |
| **RABL2B** | P12075 | chr22 | 0.094385 |
| **SURF4** | P6675 | chr9 | 0.094385 |
| **TMEM217** | P16976 | chr6 | 0.094385 |

Table S3. Significantly detected 10 obesity related TAGs in multiple regression (FDR adjusted P-value < 0.01)

| **Gene_symbol** | **Gene/Protein_ID** | **Chromosome** | **FDR_M2** |
| --- | --- | --- | --- |
| PPP1R1A | P8750 | chr12 | 0.088509 |
| SYT13 | P18875 | chr11 | 0.088509 |
| HADH | P8210 | chr4 | 0.088509 |
| IAPP | P6894 | chr12 | 0.088509 |
| LOC100129046 | NR_034091 | chr1 | 0.088509 |
| RAD9B | P6433 | chr12 | 0.088509 |
| PLCXD3 | P5584 | chr5 | 0.090744 |
| ANKRD36B | P24185 | chr2 | 0.090744 |
| FAM174B | P16809 | chr15 | 0.090744 |
| SCGN | P27914 | chr6 | 0.090744 |

Table S4. Selected representative genes included in the four categories.

| **Gene Symbol** | **DESeq2** | **Ordinary regression** | **Robust regression** | **Category** | **Data** |
| --- | --- | --- | --- | --- | --- |
| **IL6** | 0.003546 | 0.046317 | 0.026386 | **AM** | **Human RNA-seq** |
| **AMY2A** | 0.001012 | 0.219362 | 0.153705 | **EM** |
| **PLCXD3** | 0.246798 | 5.94E-05 | 0.000174 | **SM** |
| **STX6** | 1 | 0.085651 | 0.002018 | **RM** |
| **TOX4** | 2.82E-05 | 0.001313 | 0.001594 | **AM** | **Bovine RNA-seq** |
| **SECTM1** | 0.000592 | 0.299339 | 0.339181 | **EM** |
| **SAMD4A** | 0.108342 | 0.002724 | 0.0038 | **SM** |
| **PMCH** | 0.245253 | 0.255741 | 0.002533 | **RM** |

Table S5. The result of the RNA-seq analysis of 16 randomly selected representative genes across the four categories for qRT-PCR experiments.

| **Gene_Symbol** | **DESeq2** | **Ordinary regression** | **Robust regression** | **Category** |
| --- | --- | --- | --- | --- |
| **HNRNPL** | 0.000255 | 0.000301 | 0.000402 | Significant results from all methods (AM) |
| **NOS3** | 0.000154 | 0.002586 | 0.002841 |
| **SPTSSB** | 0.000132 | 0.013805 | 0.007318 |
| **TOX4** | 2.82E-05 | 0.001313 | 0.001594 |
| **FAM166B** | 0.015045 | 0.752716 | 0.78386 | Only significant results from existing methods (EM) |
| **RNPC3** | 0.023477 | 0.697128 | 0.717371 |
| **SECTM1** | 0.000592 | 0.299339 | 0.339181 |
| **SPESP1** | 0.001219 | 0.614653 | 0.657822 |
| **C25H16orf88** | 0.179103 | 0.008653 | 0.008653 | Only significant results from suggesting methods (SM) |
| **KALRN** | 0.074436 | 0.009155 | 0.01095 |
| **PRIM2** | 0.271295 | 0.01812 | 0.024116 |
| **SLC4A11** | 0.061927 | 0.024854 | 0.060003 |
| **NLN** | 0.248911 | 0.094903 | 0.013644 | Only significant results from robust method (RM) |
| **PBX4** | 0.272481 | 0.07405 | 0.004263 |
| **PMCH** | 0.245253 | 0.255741 | 0.002533 |
| **RECQL5** | 0.31051 | 0.1309 | 0.019035 |

**Supplementary Information**

**Holstein RNA-seq pipe-line**

The RNA was collected from 21 Holstein cows in their 2nd−4th lactation, raised at the Kang Sung Won farm, Korea. Cows were kept in free stall housing, fed with total mixed ration (TMR) and supplied with water *ad libitum*. They were milked twice a day, at 4 a.m. and 4 p.m., in a designated milking parlor and all Korea Hazard Analysis and Critical Control Point (HACCP) guidelines were followed. Milk samples were collected by hand-milking 2−3 hours after the evening milking at 60, 100−160, 180−210, and 240−270 days of lactation. Samples were assessed for cell viability using the typan blue method and total RNA was extracted. Somatic cells were collected from fresh milk treated with 50 μL of 0.5 M EDTA, centrifuged at 1800 rpm at 4℃ for 15 min, and washed with 10 ml of PBS (pH 7.2, diluted with 0.1% DEPC) and 10 μL of 0.5 M EDTA. Cells were centrifuged at 1800 rpm, 4 ℃ for 15 min, and re-suspended in PBS after supernatant removal. Total RNA isolation was performed according to the manufacturer instructions using the TRIzol reagent (Molecular Research Center, Cincinnati, OH, USA). Total RNA levels were quantified by absorbance at 260 nm using ND-1000 spectrophotometer (Fisher Thermo, Wilmington, MA), and RNA integrity was assessed by 1% (w/v) agarose gel electrophoresis followed by ethidium bromide staining of the 28S and 18S bands. Total RNA (1 µg amounts) was reverse-transcribed into cDNA using an iScript cDNA Synthesis kit (Bio-Rad, Hercules, CA), following the manual. All primers were designed using reference sequences published by the National Center for Biotechnology Information. Pre-processing of Holstein RNA-seq data.

After RNA extraction, we performed RNA-seq using Illumina HiSeq 2000 platform based on basic instruction. For removing adapters, we used Trimmomatic (13) with following option : PE -phred33 ILLUMINACLIP:TruSeq3-PE.fa:2:30:10 MINLEN:75 2. These clean-reads were mapped to the reference genome (BosTau7) from UCSC database using Bowtie2, included in Tophat2 (14); it is one of the most commonly used tools for mapping to the genome reference. The aligned result from the Tophat2 in BAM was converted to SAM format by SAMtools (15). After that, gene expression levels were estimated by HTseq package (16), implemented in python, with Bos taurus7 gene transfer format (.GTF) file. From the read mapping result, annotated gene expression levels were estimated by previous steps. No expressed (All zero counted genes) and correlated (Spearman correlation coefficient < 0.1) genes were pre-screened to remove redundant features. Using these data, we performed statistical test for detecting milk production related genes.
